# Tunable Bandgap in Cobalt-Doped FeS_2_ Thin Films for Enhanced Solar Cell Performance

**DOI:** 10.3390/ma18194546

**Published:** 2025-09-30

**Authors:** Eder Cedeño Morales, Yolanda Peña Méndez, Sergio A. Gamboa-Sánchez, Boris Ildusovich Kharissov, Tomás C. Hernández García, Marco A. Garza-Navarro

**Affiliations:** 1 Departamento de Materiales I, Facultad de Ciencias Químicas, Universidad Autónoma de Nuevo León, Ave. Universidad S/N, Cd. Universitaria, San Nicolás de los Garza 66455, Nuevo León, Mexico; emc220003@utdallas.edu (E.C.M.); boris.kharissov@uanl.edu.mx (B.I.K.); tomas.hernandezgr@uanl.edu.mx (T.C.H.G.); 2 Instituto de Energías Renovables, Universidad Nacional Autónoma de México, Privada Xochicalco S/N, Centro., Temixco 62580, Morelos, Mexico; sags@ier.unam.mx; 3 Facultad de Ingeniería Mecánica y Eléctrica, Universidad Autónoma de Nuevo León, Ave. Universidad S/N, Cd. Universitaria, San Nicolás de los Garza 66455, Nuevo León, Mexico; marco.garzanr@uanl.edu.mx

**Keywords:** chemical bath deposition, thin film solar cells, p-type semiconductor, band gap, pyrite structure

## Abstract

Cobalt-doped iron disulfide (FeS_2_) thin films were synthesized via chemical bath deposition (CBD) followed by annealing at 450 °C, yielding phase-pure pyrite structures with multifunctional properties. A deposition temperature of 95 °C is critical for promoting Co incorporation, suppressing sulphur vacancies, and achieving structural stabilization of the film. After annealing, the dendritic morphologies transformed into compact quasi-spherical nanoparticles (~100 nm), which enhanced the crystallinity and optoelectronic performance of the films. The films exhibited strong absorption (>50%) in the visible and near-infrared regions and tunable direct bandgaps (1.14 to 0.96 eV, within the optimal range for single-junction solar cells. Electrical characterization revealed a fourth-order increase in conductivity after annealing (up to 4.78 Ω^−1^ cm^−1^) and confirmed stable p-type behavior associated with Co^2+^-induced acceptor states and defect passivation. These results demonstrate that CBD enabled the fabrication of Co-doped FeS_2_ thin films with synergistic structural, electrical, and optical properties. The integration of earth-abundant elements and tunable electronic properties makes these films promising absorber materials for the next-generation photovoltaic devices.

## 1. Introduction

Iron disulfide (FeS_2_) has emerged as a leading earth-abundant semiconductor for photovoltaic applications owing to its optimal bandgap (0.9–1.5 eV), high absorption coefficient (>10^5^ cm^−1^), and low-cost precursors (Fe and S) [1,2]. The pyrite phase of FeS_2_ is particularly attractive for thin-film solar cells because of its superior optoelectronic properties and potential to reduce energy production costs compared to conventional materials such as silicon and CdTe [3,4,5]. However, uncontrolled sulfur vacancies often lead to undesired n-type conductivity and phase instability, thereby limiting the device efficiency.

Transition metal doping, particularly with cobalt (Co^2+^), has been explored to stabilize the phase, passivate defects, and induce p-type conductivity in doped FeS_2_ materials [6,7,8]. Recent studies on electrochemically deposited Co-doped FeS_2_ have demonstrated ferromagnetism and bandgap tunability (~0.9 eV); however, such methods face scalability challenges owing to complex setups or high-energy requirements.

Chemical bath deposition (CBD) is a simple, low-temperature, and scalable technique that allows fine stoichiometric control and uniform thin-film growth at atmospheric pressure. Despite the advantages of CBD, its application in cobalt-doped FeS_2_ thin films remains largely unexplored, particularly for achieving stable p-type behavior and desirable magnetic properties [9,10,11]. Previous attempts using other methods, such as electrochemical deposition, have had limited success in controlling sulfur vacancies and optimizing optoelectronic-magnetic synergy. The lack of studies on CBD-synthesized Co-doped FeS_2_ represents a critical opportunity, given the potential of CBD for large-area, low-cost production [12,13].

In this study, Co-doped FeS_2_ thin films were synthesized via CBD, and the effects of the deposition temperature (90 and 95 °C) and post-annealing (450 °C) on the semiconducting properties were investigated. A pure pyrite phase was observed at 95 °C with Co^2+^ incorporation, which minimized sulfur vacancies. The films exhibited tunable optoelectronic properties, including a reduced bandgap after annealing and enhanced p-type conductivity, which were achieved through the Co^2+^-induced acceptor states. These results demonstrate the feasibility of preparing multifunctional Co-doped FeS_2_ films that combine optoelectronic properties for use in next-generation solar cells.

## 2. Materials and Methods

### 2.1. Thin Film Deposition

Co-doped FeS_2_ thin films were synthesized by chemical bath deposition (CBD) using high-purity reagents: ferrous ammonium sulfate hexahydrate (Fe(NH_4_)_2_(SO_4_)_2_-6H_2_O, 99.9%), sodium thiosulfate (Na_2_S_2_O_3_, 99.4%), and cobalt (II) sulfate heptahydrate (CoSO_4_-7H_2_O, 98.0%). Standard borosilicate glass slides (Corning, NY, USA) were used as substrates. Prior to deposition, the slides were cleaned sequentially, rinsed with alkaline detergent and distilled water, and immersed in deionized water for 3 min, followed by ultrasonication in acetone and methanol for 10 min each. A final rinse with deionized water removed impurities.

The chemical bath was prepared by mixing 16 mL of 0.5 M Fe(NH_4_)_2_(SO_4_)_2_-6H_2_O, 20 mL of 1 M Na_2_S_2_O_3_, 8 mL of 0.02 M CoSO_4_-7H_2_O, and 6 mL of deionized water. The substrates were vertically immersed in a bath and maintained at either 90 or 95 °C for 3.5 h using a VWR 1150S reflux system (VWR International, Radnor, PA, USA). After the deposition, uniform dark films were observed on the substrates. Non-adherent materials and residues were removed using a dilute acid wash. All samples were annealed in a high-vacuum furnace (VBF Model 1200X, Hefei Kejing Material Technology Co., Ltd., Hefei, China) at 450 °C and 9 × 10^−3^ Torr for 1 h to enhance their crystallinity. The final film thickness, measured using an Alpha Step profilometer (Tencor KLA D-100, KLA corporation, Milpitas, CA, USA), was less than 150 nm in all cases.

### 2.2. Characterization

Crystalline phase identification was performed via X-ray diffraction (XRD) using a Rigaku D-max 2000 diffractometer (Rigaku Corporation, Tokyo, Japan) with Cu Kα radiation (λ = 1.5406 Å). Scans were performed in the 2θ range of 20–70°, and the crystallite size was calculated using the Scherrer equation based on the (200) diffraction peak.

The surface topography was assessed using atomic force microscopy (AFM, Advanced Angstrom AA3000, Angstrom Advanced Inc., Stoughton, MA, USA), and the morphological characteristics and particle size distributions were investigated using field-emission scanning electron microscopy (FESEM, Hitachi S5500, Hitachi, Ltd., Tokyo, Japan and JEOL JSM-6701F, JEOL Ltd., Tokyo, Japan, respectively). The elemental composition was determined using energy-dispersive X-ray spectroscopy (EDS).

Optical transmittance and reflectance spectra were recorded using a JASCO V-770 UV-Vis-NIR spectrophotometer (JASCO Corporation, Tokyo, Japan) in the range of 190–3100 nm. The bandgap energy (Eg) values were extracted from Tauc plots constructed from the absorption coefficient (α) versus photon energy (hν), assuming a direct bandgap transition.

The electrical properties were characterized using a Keithley 6487 picoammeter/voltage (Keithley Instruments, Cleveland, OH, USA) source connected to silver paste contacts (3 × 3 mm^2^) applied to the surface of the film. The photoresponse was measured at a 10 V bias over 60 s cycles: 20 s in the dark, 20 s under illumination, and 20 s back in the dark.

## 3. Results and Discussion

### 3.1. Structural Characterization by XRD Measurements

**Figure 1:**
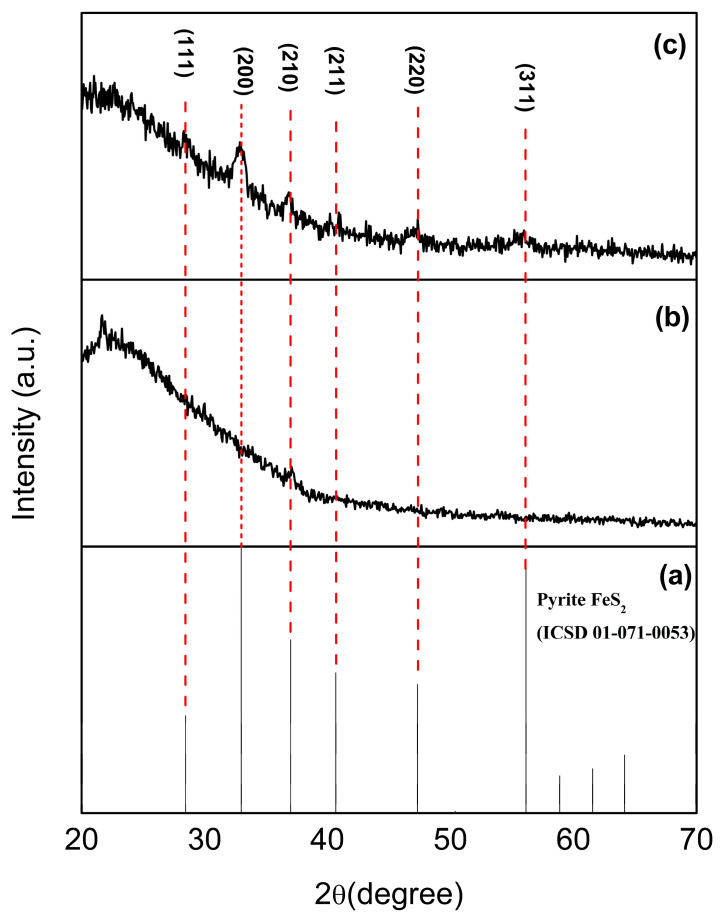
XRD patterns of Co^2+^ doped FeS_2_ thin films thermally treated at 450 °C for 1 h. (**a**) ICSD pattern of pyrite FeS_2_, (**b**) prepared at 90 °C, and (**c**) prepared at 95 °C.

The crystalline structures of the Co^2+^-doped FeS_2_ thin films were investigated using X-ray diffraction (XRD) after annealing at 450 °C. Figure 1a shows the theoretical diffraction pattern of pyrite (FeS_2_, which serves as a reference for phase identification). As shown in Figure 1b, the film synthesized at 90 °C does not exhibit any characteristic peaks associated with the pyrite phase. This suggests the formation of either nanocrystalline or pseudocrystalline materials with limited cobalt incorporation, probably owing to the insufficient solubility of Co^2+^ ions at this lower deposition temperature.

In contrast, the film deposited at 95 °C and subjected to the same annealing process, as shown in Figure 1c, exhibited distinct diffraction peaks at 32.42°, 36.46°, and 40.12° on the 2θ axis. These peaks corresponded to the (200), (210), and (211) crystallographic planes of the cubic pyrite structure, indicating successful phase formation. Although peaks were present, they exhibited broadened and moderate-intensity characteristics of the nanocrystalline domains. A slight negative shift (~0.2°) in the 2θ positions of these peaks is also observed, indicating lattice distortion in the sample structure. This distortion is attributed to the substitution of Fe^2+^ (ionic radius: 0.78 Å) with slightly smaller Co^2+^ ions (0.74 Å), which leads to strain in the crystal lattice structure. The absence of secondary phase peaks confirms that Co^2+^ incorporation occurred without segregation into distinct cobalt-based phases.

The average crystallite size was calculated to be approximately 10 nm by applying the Scherrer equation (Equation (1)) to the (200) diffraction peak [14].

(1) $$D=\frac{kl}{b\;Cosq}$$

where *D* is the average crystallite size, *l* is the X-ray wavelength, *b* is the full width at half maximum (FWHM) of the diffraction peak in radians, *k* is the Scherrer constant, which depends on the shape of the crystallite and the method used to calculate the size, and θ is the Bragg’s angle.

This nanometric size explains the peak broadening observed in Figure 1c and is consistent with the expected structural properties of thin films of this thickness (~100–120 nm). The small domain size combined with the peak shift further supports the hypothesis of cobalt-induced lattice deformation.

The incorporation of Co^2+^ ions appears to enhance the stability of the pyrite phase by improving the structural ordering and minimizing sulfur vacancies, which are considered native defects that influence the electronic behavior of FeS_2_-based semiconductors. The coordination of cobalt with S^2−^ anions may play a role in neutralizing these vacancies, thereby facilitating the formation of phase-pure pyrite.

These results demonstrate that a small increase in the deposition temperature can significantly affect the crystallization pathway of FeS_2_ during CBD. The optimal conditions found at 95 °C enhanced Co^2+^ solubility and lattice incorporation, enabling the formation of structurally stable phase-pure pyrite with nanoscale grain sizes and properties suitable for photovoltaic applications [15].

### 3.2. Thickness and Surface Analysis

The thicknesses and surface morphologies of the Co^2+^-doped FeS_2_ thin films before and after thermal annealing were systematically analyzed using atomic force microscopy and profilometry. These characterizations are essential for understanding how the deposition temperature and post-synthesis treatment influence film uniformity, roughness, and structural consolidation, all of which are critical for optimizing semiconductor performance in photovoltaic applications.

**Figure 2:**
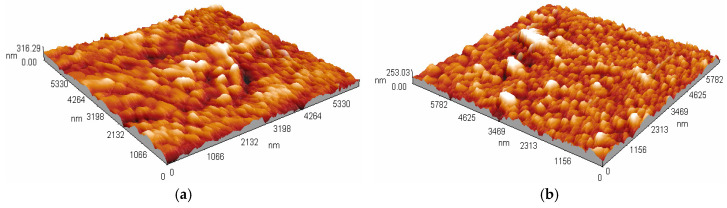
AFM images of Co^2+^ doped FeS_2_ thin films deposited at (**a**) 90 °C, and (**b**) 95 °C.

**Figure 3:**
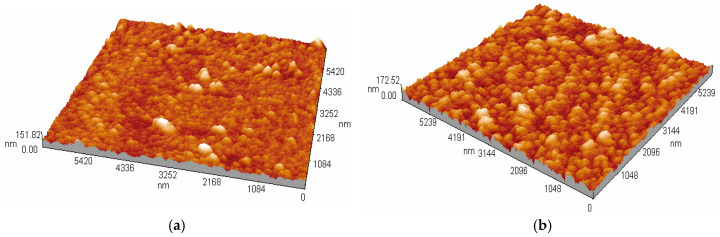
AFM images of Co^2+^ doped FeS2-based thin films thermally treated at 450 °C for 1 h and originally prepared at: (**a**) 90 °C, and (**b**) 95 °C.

Analysis of images obtained using atomic force microscopy (AFM) enables precise assessment of the surface morphology of thin films, which is crucial for understanding their performance in electronic and optoelectronic applications. Co-doped FeS_2_ thin films deposited at 90 °C and 95 °C, both before and after heat treatment at 450 °C for one hour, were examined using AFM in contact mode. The analysis was conducted over areas of 5.33 × 5.33 μm^2^ for Figure 2a and Figure 2b and 5.42 × 5.42 μm^2^ and 5.239 × 5.239 μm^2^ for Figure 3a and Figure 3b, respectively, to minimize edge effects. The free and open-source software Gwyddion version 2.69 was used to analyze the AFM images and evaluate the surface roughness. As shown in Figure 2a, the topography of the film deposited at 90 °C without heat treatment exhibited a structure of great interest. The image displays dendritic structures with a heterogeneous distribution and pronounced surface roughness. Quantitative analysis indicated a mean quadratic roughness (Rq) of 80.02 nm, calculated from the standard deviation of the elevation points and valleys of the relative height variations in the ridges. This value aligns with the observed visual roughness and indicates a high density of surface defects, such as sulfur vacancies, which can influence the charge carrier mobility. Figure 2b shows the film deposited at 95 °C without heat treatment. The topography displays a more uniform surface with well-spread, hemispherical nanostructures. This morphology indicates more controlled nucleation and a higher density of growth centers, which, according to the specialized literature, is linked to a greater degree of structural order in FeS_2_ films [16]. The roughness value Rq recorded was 55.19 nm, representing a significant decrease compared to that of the sample deposited at 90 °C. This outcome reflects a notable reduction in surface irregularities, suggesting improved precursor diffusion at higher temperatures during synthesis. When heat treatment was applied at 450 °C, significant changes in the topography occurred. Figure 3a, which shows the treated sample originally deposited at 90 °C, displays a transformation of the dendritic structures into a denser aggregation of nearly spherical nanoparticles. The roughness Rq was lowered to 42.16 nm, suggesting that heat treatment likely encourages surface reorganization and the correction of growth defects through the diffusion of Co^2+^ and the rearrangement of the crystal lattice, which stabilizes the pyrite phase and reduces sulfur vacancies. Figure 3b shows the heat-treated film deposited at 95 °C. A dense, uniform surface composed of compact nanoparticles was observed. Analysis of the samples showed that the roughness Rq of the tested sample was the lowest, at 33.08 nm. This indicates a highly consistent surface, likely resulting from the correct deposition temperature combined with thermal recrystallization. The results suggest that the complete diffusion of Co^2+^ ions occurs, which helps stabilize the structure and passivate surface defects such as sulfur vacancies. This, in turn, enhances the optoelectronic properties of the materials. The analysis of the roughness of the FeS_2_ thin films revealed specific features related to the synthesis time and heat treatment, providing more detailed and quantitative insights. It should be noted that Rq is sensitive to the overall height distribution and allows for a more precise correlation between the structural changes caused by the synthesis temperature and heat treatment.

The thickness measurements obtained by stylus profilometry showed average values of approximately 100 nm for the annealed film deposited at 90 °C, and approximately 120 nm for the film deposited at 95 °C. These values are in good agreement with the relative height distributions observed by AFM and confirm that both films remained thin after thermal treatment.

The improved surface morphology of the annealed films is highly beneficial for optoelectronic devices [17]. Smoother and more homogeneous surfaces reduce the interfacial resistance at the layer junctions, improving the electrical contact between the semiconductor and the adjacent conductive or buffer layers. In addition, uniform nanoparticle packing facilitates carrier mobility and collection, which are essential for efficient current extraction in photovoltaic systems. These morphological improvements also correlate with the structural results described in Section 3.1, where the film deposited at 95 °C exhibited superior crystallinity and clear formation of the pyrite phase. The combination of reduced surface roughness, increased structural order, and preservation of thin-film dimensions strongly supports the conclusion that deposition at 95 °C followed by annealing at 450 °C is the optimal synthesis route for the preparation of high-quality Co^2+^-doped FeS_2_ thin films suitable for integration into solar energy conversion devices.

### 3.3. Morphology and Surface Composition Analysis

This study was conducted to investigate the morphological evolution and elemental composition of Co^2+^-doped FeS_2_ thin films synthesized at 95 °C. The results showed that the samples deposited at 95 °C exhibited superior structural and surface properties compared with those deposited at 90 °C. Field-emission scanning electron microscopy and energy-dispersive X-ray spectroscopy were used in this study. Analyses were performed both before and after thermal annealing at 450 °C to evaluate the effects of the heat treatment on the surface structure and compositional homogeneity of the films.

**Figure 4:**
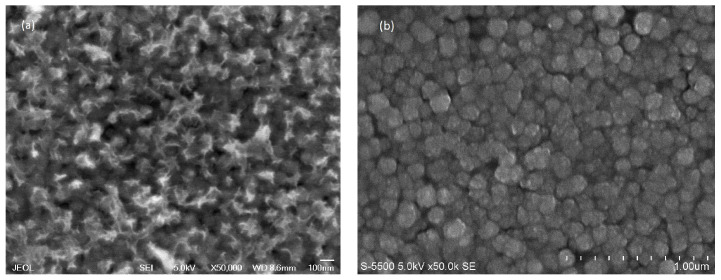
FESEM micrographs of Co^2+^ doped FeS_2_ thin film: (**a**) as deposited at 95 °C and (**b**) at 450 °C for 1 h.

As shown in Figure 4a, the surface morphology of the as-deposited film is characterized by dendritic nanoparticle clusters uniformly distributed over the substrate. These branched structures are typical of the non-equilibrium growth processes commonly observed in chemical bath deposition, particularly under conditions of high supersaturation and rapid ionic aggregation. The dendritic configuration implies a relatively open structure with a high surface-to-volume ratio and considerable surface roughness, which is consistent with the topographical features observed by AFM. The presence of these dendritic aggregates suggests that the crystal growth was kinetically favored in certain crystallographic directions at the time of deposition, resulting in the formation of elongated tree-like nanostructures.

Thermal annealing at 450 °C induced a pronounced change in surface morphology, as shown in Figure 4b. The dendritic clusters underwent structural reorganization, resulting in dense clusters of quasi-spherical nanoparticles with an average diameter of approximately 100 nm [18]. This morphological change is indicative of a solid-state recrystallization process in which atoms gain sufficient energy to overcome the initial growth anisotropies and rearrange into a more thermodynamically stable and compact structure. The reduction in surface energy from this transformation promotes grain consolidation and particle densification, thereby improving interparticle connectivity and uniformity throughout the film. This new morphology represents a more ordered and electronically favorable structure, with potential implications for improved charge transport and reduced trap-state density.

Complementary EDS analysis of the annealed film confirmed the presence of cobalt, iron, and sulfur, with a quantitative composition of 0.16. % Co, 35.28 at. % Fe, and 64.56 at. % S. This composition yielded a near-stoichiometric Fe:S ratio of 1.0:1.8, which was considered suitable for identifying the material as cobalt-doped pyrite-type FeS_2_. The presence of Co in the film after annealing, even at minimal levels, confirmed its integration into the host lattice without the formation of separate phases, as corroborated by the XRD analysis described in Section 3.1.

Based on the results from the EDS analysis, it is concluded that the Fe:S composition ratio is 1.83:0.0045. Although this ratio is not exactly stoichiometric, it is very close to the values reported for pyrite (Fe:S in the range 1:1.9 to 1:2.08) [19], as confirmed by the XRD results from all the samples prepared from this material. It is possible that the Fe and S ratio in this study influences the diamagnetic or ferromagnetic transition properties of pyrite more than its structural properties. Therefore, the samples prepared here are considered thin films with structural characteristics similar to cobalt-doped iron disulfide (Co^2+^ doped FeS_2_).

The thermally assisted diffusion of Co^2+^ ions within the Fe-S lattice appears to play a critical role in the defect passivation and structural stabilization of the crystalline lattice. The mobility of Co atoms at elevated temperatures enhances their ability to interact with native sulfur vacancies, effectively neutralizing intrinsic point defects. This interaction diminishes the chances of recombination center formation, which would otherwise impair the electronic performance. In addition, the presence of Co^2+^ helps stabilize the pyrite phase by promoting local ordering and suppressing polymorphic transformations or precipitation of cobalt-rich secondary phases. The dual effects of annealing (morphological densification and compositional optimization) resulted in a thin film with improved structural coherence and enhanced phase purity.

The microstructural consolidation observed in Figure 4b, combined with the favorable elemental distribution and stoichiometry confirmed by EDS, underscores the importance of both deposition temperature and post-deposition heat treatment. The compact morphology, increased film density, and minimized defect population achieved by this thermal protocol are expected to enhance optoelectronic properties by facilitating efficient charge transport and reducing recombination losses. These features are essential for advancing the integration of co-doped FeS_2_ films into photovoltaic devices.

### 3.4. Optical Properties and Bandgap Calculation

The optical properties of Co^2+^-doped FeS_2_ thin films were investigated using transmittance, reflectance, and bandgap measurements to evaluate their potential for photovoltaic applications. These analyses were performed before and after thermal annealing to determine how the synthesis temperature and post-deposition treatment affected the light-matter interaction, energy absorption, and optical bandgap tuning.

**Figure 5:**
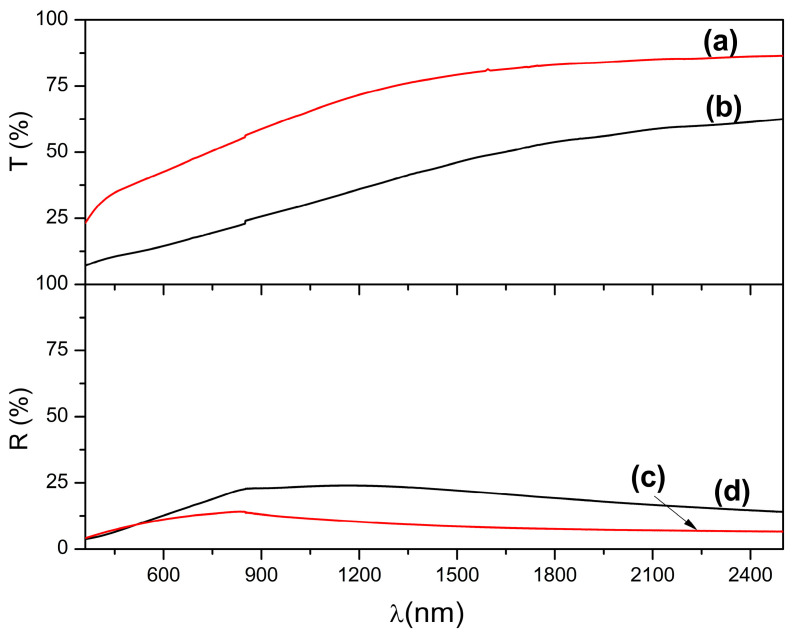
Transmittance reflectance spectra for Co^2+^ doped FeS_2_ thin films deposited (a) at 90 °C and (b) at 95 °C. Reflectance spectra for Co^2+^ doped FeS_2_ thin films deposited (c) at 90 °C and (d) at 95 °C.

Figure 5 shows the transmittance and reflectance spectra of the as-deposited films prepared at 90 and 95 °C. The film deposited at 90 °C exhibited the highest transmittance, reaching approximately 80% at 1500 nm, as shown in Figure 5a. This high transmittance indicates poor film consolidation, low absorption, and possibly incomplete pyrite phase formation, which is consistent with the less crystalline structure identified by XRD. In contrast, the film deposited at 95 °C, as shown in Figure 5b, had a significantly reduced transmittance of approximately 50%, indicating a higher optical absorption. This reduction was attributed to the presence of a denser pyrite phase and greater film thickness, as confirmed by the morphological characterization.

The reflectance spectra for both samples remained below 20%, with the film deposited at 95 °C showing slightly higher reflectance (Figure 5c) than that of the film deposited at 90 °C (Figure 5d). This difference may be due to the formation of surface Co^2+^–S^2−^ coordination near the metallic regions, which slightly increases the optical reflectance. However, the most notable difference is the absorption performance: the film prepared at 95 °C absorbs approximately 40% of the incident light compared to only 10% for the film prepared at 90 °C. This increase in light absorption is consistent with the improved crystallinity, increased morphological compactness, and slightly greater thickness of the 95 °C film (~125 nm vs. ~100 nm), which together improve the optical density and absorption depth of the film.

**Figure 6:**
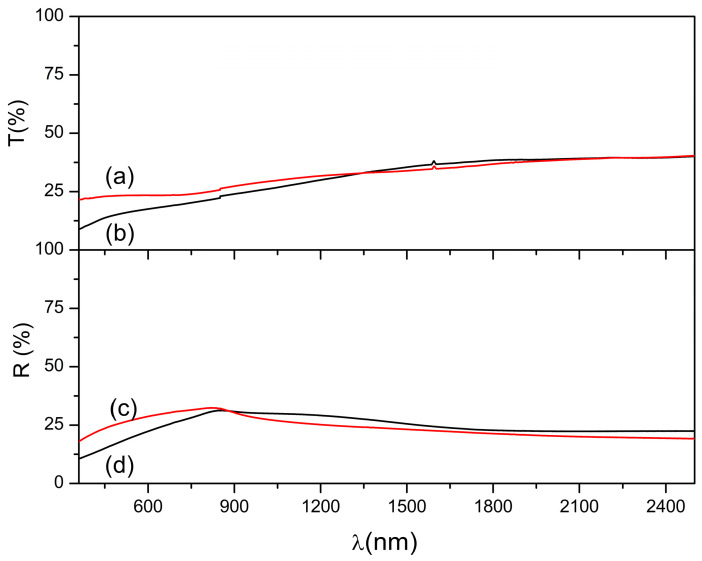
Transmittance spectra for Co^2+^ doped FeS_2_ thin films thermally treated at 450 °C for 1 h, previously synthesized at (a) 90 °C and (b) 95 °C. Reflectance spectra for the samples previously synthesized at (c) 90 °C and (d) 95 °C.

After annealing at 450 °C, the optical properties of both the films converged significantly, as shown in Figure 6. The transmittance spectra of the films annealed at 90 and 95 °C are shown in Figure 6a and Figure 6b, respectively, and the corresponding reflectance spectra are shown in Figure 6c and Figure 6d, respectively. At 1500 nm, both samples exhibited a transmittance of approximately 30% and reflectance of approximately 20%, indicating an average absorption efficiency of at least 50% across the visible and near-infrared spectra. This convergence suggests that annealing-induced structural reorganization and phase consolidation favour the pyrite phase. While the bulk optical responses appear similar, subtle variations in the local crystallinity, Co-S coordination, sulfur vacancy concentration, and nanoparticle packing density can lead to measurable differences in the electrical and electronic behavior.

**Figure 7:**
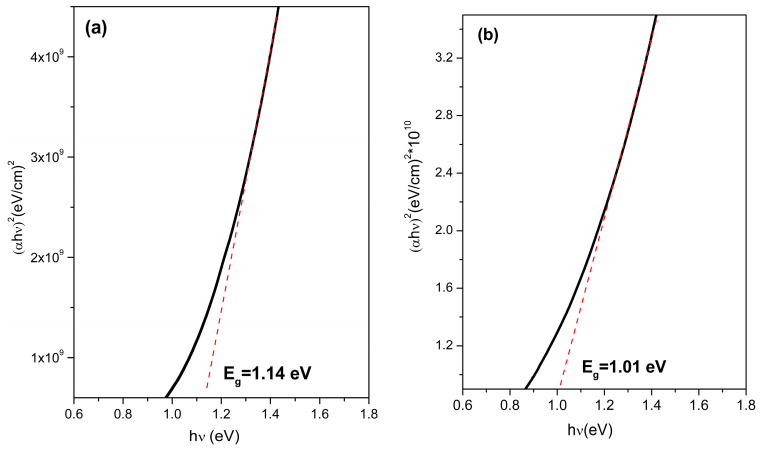
Band gap of Co^2+^ doped FeS_2_ thin films: (**a**) as deposited at 90 °C and (**b**) thermally treated at 450 °C for 1 h.

**Figure 8:**
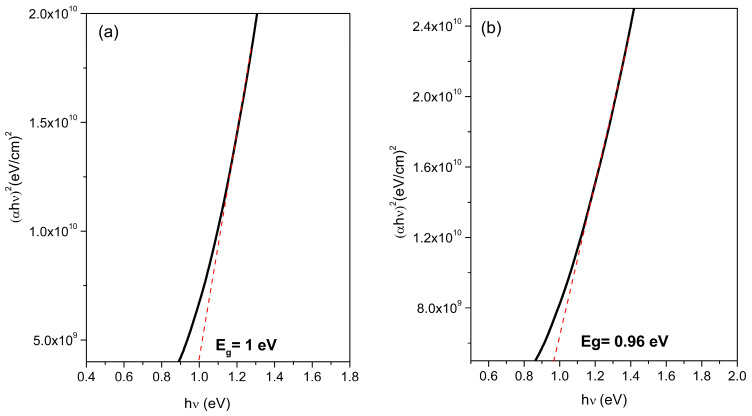
Band gap of Co^2+^ doped FeS_2_ thin films: (**a**) as deposited at 95 °C and (**b**) thermally treated at 450 °C for 1 h.

The optical bandgap energy (Eg) of the films was calculated using the Tauc plots based on the absorption coefficient derived from the combined transmittance and reflectance data. A direct transition model with a power factor of 2 was used for the analyses. The as-deposited film synthesized at 90 °C exhibited an Eg of 1.14 eV (Figure 7a), which decreased to 1.01 eV after thermal treatment (Figure 7b). A similar trend was observed for the 95 °C film, where the bandgap decreased from 1.00 eV in the as-deposited state (Figure 8a) to 0.96 eV after annealing (Figure 8b).

This consistent bandgap narrowing is attributed to annealing-induced recrystallization processes that enhance long-range order, minimize structural disorder, and reduce the density of the localized electronic states near the band edges. The observed bandgap values (ranging from 1.14 to 0.96 eV) were in or near the optimal window for single-junction solar cells [20]. The Shockley–Queisser limit describes the maximum theoretical efficiency for single-junction solar cells, which is around 1.35 eV under standard illumination conditions. Recent research shows that the development of single-junction solar cells continues to progress toward this limit [21]. To go beyond the Shockley–Queisser limit, alternative methods like multi-junction devices are necessary. Additionally, a pyrite-type semiconductor with a band gap between 1.1 and 1.4 eV could offer a good balance between open-circuit voltage and light absorption.

The film annealed at 95 °C exhibits a slightly lower energy bandgap than that in the optimal range. However, it is important to relate this result to the potential conductivity of the material and its ferromagnetic response to electronic transport across the insulator-metal transition in Co-doped pyrite. These properties make it a promising candidate for multifunctional solar energy applications [22,23,24].

Overall, the optical analyses confirmed that both the deposition temperature and annealing are critical parameters for tuning the absorption profile and electronic transitions of Co^2+^-doped FeS_2_ films. The improved absorption, bandgap tunability, and structural transformation support the integration of these materials into thin-film photovoltaic systems, where high absorption efficiency and tunable electronic properties are essential requirements.

### 3.5. Electrical Measurement

**Figure 9:**
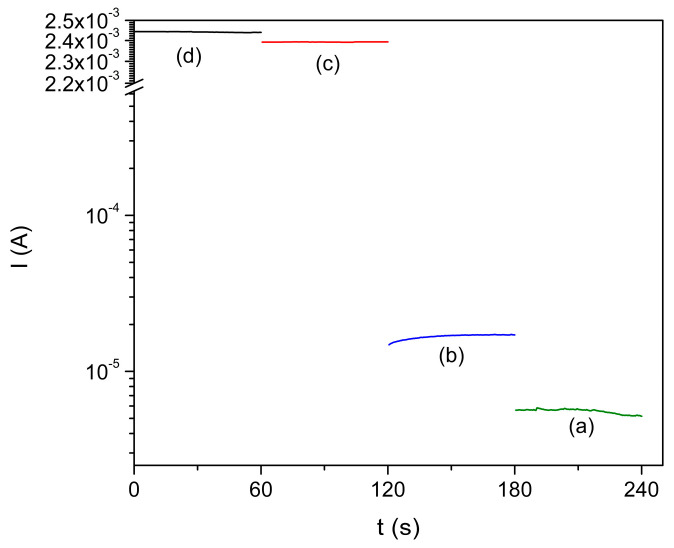
Photo-response of Co^2+^ doped FeS_2_ thin films: (a) as-deposited at 90 and (b) as-deposited at 95 °C. Thermally treated at 450 °C for 1 h: (c) deposited at 90 °C and (d) deposited at 95 °C.

The electrical properties of the Co^2+^-doped FeS_2_ thin films were evaluated using conductivity measurements and light–dark cycling photoresponse analysis to determine their viability as absorber layers in photovoltaic devices. The influence of the synthesis temperature and thermal annealing on the charge transport dynamics was thoroughly investigated, as shown in Figure 9.

The photoresponse curves show a significant difference in the electrical behavior of the as-deposited and annealed films. Figure 9a,b show the current-time profiles of the films synthesized at 90 °C and 95 °C, respectively, in their as-deposited states. Both samples exhibited low and relatively flat photocurrent responses, indicating the poor generation and collection of photogenerated carriers. These results are attributed to the limited crystallinity and presence of a high density of structural defects, such as sulfur vacancies, which are known to act as recombination centers that suppress carrier mobility and lifetimes.

After annealing at 450 °C for one hour, a dramatic improvement in the photoresponse was observed, as shown in Figure 9c,d. The photocurrent increased by almost two orders of magnitude in both cases, demonstrating that the thermal treatment was essential for activating the optoelectronic properties of the material. This enhancement was mainly due to the structural consolidation and defect passivation induced by annealing, which reduced the grain boundary resistance, improved the crystalline quality, and facilitated the carrier separation and mobilization.

Among the annealed samples, the film initially deposited at 95 °C (Figure 9d) exhibited a stronger photocurrent than that of the sample deposited at 90 °C (Figure 9c). This difference reflects the cumulative effect of the higher deposition temperature and subsequent annealing, which together promote more complete formation of the pyrite phase and better grain connectivity, as confirmed by the XRD and FESEM analyses discussed in the preceding sections. The improved photoresponse of the 95 °C film suggests that even modest increases in the synthesis temperature can yield significant improvements in the final electronic behavior of the material, particularly when followed by thermal postprocessing.

The quantitative conductivity measurements supported these photoresponse data. The electrical conductivities of the film deposited at 95 °C and the annealed reached approximately 4.78 Ω^−1^ cm^−1^. By contrast, the conductivity of the as-deposited counterpart was approximately 7 × 10^−3^ Ω^−1^ cm^−1^, indicating an increase of nearly four orders of magnitude after annealing. This significant increase in electrical conductivity was consistent with the observed improvements in crystallinity, phase purity, and nanoparticle compaction [25]. This also confirms the reduction in the number of charge-trapping sites, particularly sulfur vacancies, which would otherwise limit the carrier transport.

Complementary hot-probe measurements confirmed the p-type nature of the annealed films regardless of the deposition temperature. This p-type behavior results from the formation of native acceptor states within the crystal lattice, promoted by the substitution of Fe^2+^ ions with Co^2+^ ions. The smaller ionic radius of Co^2+^ leads to local lattice deformation and the generation of holes as majority carriers, which enables p-type conductivity. The ability to reliably induce p-type behavior in FeS_2_ through controlled cobalt doping is a long-standing challenge in this material system, which often exhibits unwanted n-type conductivity owing to intrinsic defects in the crystal structure.

These electrical results confirm that the processing parameters, particularly the combination of deposition at 95 °C and annealing at 450 °C, optimize the material for photovoltaic applications. The significantly improved conductivity, stable p-type character, and enhanced photocurrent response validate the Co^2+^-doped FeS_2_ thin films as strong candidates for use as absorber layers in heterojunction solar cells. The p-type nature of the material is particularly important for establishing a built-in electric field when paired with an n-type contact, which enables the efficient charge separation and extraction of the generated carriers. The observed improvements in the optoelectronic behavior reflect the synergy between the morphological refinement, structural order, and compositional control enabled by this optimized processing route.

### 3.6. Magnetic Properties

**Figure 10:**
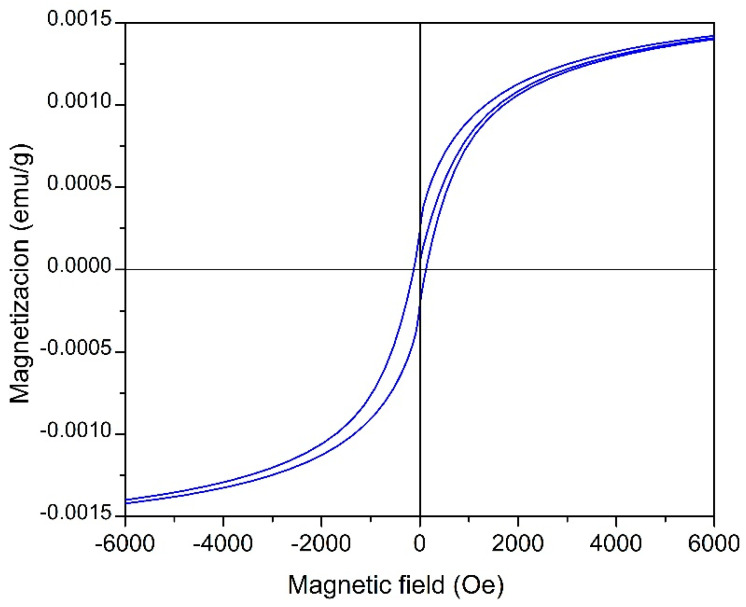
Magnetic hysteresis at 300 K for the Co^2+^ doped FeS_2_ thin films deposited at 95 °C, thermally treated at 450 °C for 1 h.

The magnetic response of FeS_2_ thin films doped with Co^2+^ ions exhibited low magnetization values at 300 K, as shown in Figure 10. These diminished values align with the very dilute incorporation of cobalt (approximately 0.16 at %) and the propensity of Co^2+^ ions to stabilize in a low-spin state within the pyrite crystal lattice, which restricts the effective magnetic moment per substituted site [24,25]. Furthermore, the observed sulfur deficiency (S/Fe ratio of approximately 1.83) indicates that the films deviate from the ideal stoichiometry, leading to the introduction of sulfur vacancies that serve as defect states. This compositional variation alters the carrier density and local exchange pathways, ultimately weakening the long-range magnetic order and reducing the macroscopic magnetization.

According to previous studies, the substitution of Fe with Co in FeS_2_ leads to the formation of narrow electronic states near the conduction band edge. This phenomenon promotes Stoner-type instabilities and facilitates weak ferromagnetic responses at low carrier concentrations [26]. Conversely, sulfur deficiency and vacancy formation have been demonstrated to exert a substantial influence on magnetic coupling, potentially situating the system near a transition between non-magnetic and diluted ferromagnetic states [26,27]. Consequently, the response of the films may be positioned near the microphase boundary. This means that slight modifications in the stoichiometric composition or doping concentration could alter the equilibrium between weak ferromagnetic and nonferromagnetic behaviors. This phenomenon may offer a potential explanation for the low magnetization hysteresis loops observed in this study.

Therefore, the low magnetization values shown in Figure 10 should not be misinterpreted as inaccuracies but rather as inherent properties of the thin films of FeS_2_ doped with Co in a diluted state. This phenomenon will be explored in future research, which will use repeated magnetic measurements to better understand the interactions among stoichiometry, defects, and magnetism in Co^2+^-doped FeS_2_-based thin films for photovoltaic applications.

## 4. Conclusions

Co^2+^-doped FeS_2_ thin films with thicknesses below 150 nm were successfully synthe sized via chemical bath deposition (CBD) followed by annealing at 450 °C. The structural and functional attributes of the materials were significantly affected by the deposition temperature. Phase-pure pyrite FeS_2_ was obtained at 95 °C, as confirmed by X-ray diffraction and EDS. Annealing facilitated Co^2+^ diffusion, minimized sulfur vacancies, and induced morphological reorganization into quasi-spherical nanoparticles (~100 nm). Optical characterization revealed a tunable direct bandgap ranging from 1.14 to 0.96 eV, with annealed films absorbing over 50% of visible light. These values are within the optimal range of single-junction solar cells. The electrical conductivity increased by four orders of magnitude after annealing, reaching 4.78 Ω^−1^ cm^−1^ for the film deposited at 95 °C. Hot-probe measurements confirmed stable p-type conductivity owing to Co^2+^-induced acceptor states and defect passivation. This study demonstrated that CBD enables the fabrication of multifunctional Co-doped FeS^2^ thin films with tunable optoelectronic and magnetic properties. The simultaneous control of the bandgap, conductivity, and magnetism using a scalable and inexpensive method makes these films strong candidates for future photovoltaic and spintronic applications. However, further research is necessary to enhance our understanding of the impact of the chemical composition on the magnetic properties of these thin films. This knowledge is crucial for advancing the application of these materials in solar cells and optoelectronic devices.

## Data Availability

The original contributions presented in this study are included in the article. Further inquiries can be directed to the corresponding author.
